# *GhASHH1.A* and *GhASHH2.A* Improve Tolerance to High and Low Temperatures and Accelerate the Flowering Response to Temperature in Upland Cotton (*Gossypium hirsutum*)

**DOI:** 10.3390/ijms252011321

**Published:** 2024-10-21

**Authors:** Jisheng Ju, Junning Yang, Jiazhi Wei, Wenmin Yuan, Ying Li, Dandan Li, Pingjie Ling, Qi Ma, Caixiang Wang, Maohua Dai, Junji Su

**Affiliations:** 1 Gansu Provincial Key Laboratory of Aridland Crop Science, College of Life Science and Technology, Gansu Agricultural University, Lanzhou 730070, China; 18893855307@126.com (J.J.); jnyang01@126.com (J.Y.); wjzgsau@126.com (J.W.); yuanwm2022@163.com (W.Y.); 17834316987@163.com (Y.L.); m15293180496@163.com (D.L.); linger12012021@163.com (P.L.); wangcaix@gsau.edu.cn (C.W.); 2 Cotton Research Institute, Xinjiang Academy of Agricultural and Reclamation Science, Shihezi 832000, China; xjnkymaqi1123@163.com; 3 Hebei Provincial Key Laboratory of Crop Drought Resistance Research, Institute of Dryland Farming, Hebei Academy of Agriculture and Forestry Sciences, Hengshui 053000, China

**Keywords:** upland cotton, temperature, virus-induced gene silencing (VIGS), temperature stress, flowering time

## Abstract

The trithorax group (TrxG) complex is an important protein in the regulation of plant histone methylation. The ABSENT, SMALL, OR HOMEOTIC DISCS 1 (ASH1) gene family, as important family members of the TrxG complex, has been shown to regulate tolerance to abiotic stress and growth and development in many plants. In this study, we identified nine *GhASH1s* in upland cotton. Bioinformatics analysis revealed that *GhASH1s* contain a variety of cis-acting elements related to stress resistance and growth and development. The transcriptome expression profiles revealed that *GhASHH1.A* and *GhASHH2.A* genes expression were upregulated in flower organs and in response to external temperature stress. The results of virus-induced gene silencing (VIGS) indicated that *GhASHH1.A* and *GhASHH2.A* genes silencing reduced the ability of cotton to adapt to temperature stress and delayed the development of the flowering phenotype. We also showed that the silencing of these two target genes did not induce early flowering at high temperature (32 °C), suggesting that *GhASHH1.A* and *GhASHH2.A* might regulate cotton flowering in response to temperature. These findings provide genetic resources for future breeding of early-maturing and temperature-stress-tolerant cotton varieties.

## 1. Introduction

Temperature is an essential factor for plant growth and development and plays a crucial role in key physiological processes, such as nutrient absorption, photosynthesis, respiration, plant growth, and reproduction [1,2]. Excessively high and excessively low temperatures can harm plants; for example, high temperatures can cause plant leaves to wilt, dry out, and even fall off, whereas low temperatures can slow plant metabolism, consequently impacting plant growth and development [3,4]. Since plants are immobile organisms, they cannot relocate to more favorable environments when faced with extreme temperatures. Fortunately, plants can adjust their metabolic processes in response to environmental factors, altering their growth patterns [5]. This adaptive mechanism allows plants to cope effectively with harsh or stressful environments and to safeguard their delicate growth stages from unfavorable conditions [6]. Chromatin modification is involved in the transcriptional regulation of stress-related genes to modulate the stress response of plants so that they can quickly adapt to environmental temperature fluctuations. It also regulates temperature-dependent flowering and thermomorphogenesis and plays an important regulatory role in the process of plant adaptation and evolution [7,8]. In plants, chromatin modifiers are important during development and in response to environmental change.

The chromatin modifiers Polycomb Group (PcG) and Trithorax Group (TrxG) are two protein complexes that play crucial roles in regulating transcriptional repression or activation. The former functions primarily through histone lysine methyltransferase (HKMT) activity to repress gene expression, whereas the latter mainly activates transcription through similar enzymatic mechanisms [9]. The PcG protein complex acts on the polycomb repressive complex (PRC), binding to target genes and integrating H3K27me3 to inhibit gene expression [10]. *TrxG*, an antagonistic gene of PcG, activates gene expression through the integration of H3K4me3 and H3K36me3 [11]. In addition, PcG and TrxG play important roles in plant plasticity by regulating homeotic gene transcription in vivo and participating in developmental processes in response to environmental signals [12]. TrxG also regulates stress responses and is involved in intergenerational memory [13]. During low-temperature exposure, *Arabidopsis* regulates flowering by increasing PRC2 deposition of H3K27me3 on *FLOWERING LOUCS C* (*FLC*) chromatin to inhibit its expression [14]. After heat stress, *constitutive photomorphogenesis 5A* (*CSN5A*) regulates heat stress memory by increasing H3K4me3 abundance in the memory genes *ASCORBATE PEROXIDASE 2* (*APX2*) and *HEAT SHOCK PROTEIN 22* (*HSP22*) [15]. In plants, the functions and mechanisms of PcG have been well studied. However, few studies regarding those of TrxG have been reported [16].

The ABSENT, SMALL, OR HOMEOTIC DISCS 1 (ASH1) family is an important class of genes belonging to the TrxG complex, and it catalyzes the trimethylation of H3K36 and is also involved in H3K4 methylation [16,17]. ASH1 antagonizes PcG-mediated silencing via H3K36 dimethylation at *homeobox* (*HOX*) genes [18]. In *Arabidopsis thaliana*, five *ASH1*s have been identified, and the *ASH1* gene family includes the *ASHH1*, *ASHH2, ASHH3*, and *ASHR3* subfamilies. Previous studies on *ASHH1* have focused mainly on the regulation of plant flowering; in addition, ASHH1 is involved in UV-B damage and DNA repair [19]. *ASHH2* is a major di- and tri-methyltransferase for H3K36 and is related to dehydration stress, plant defense against fungal pathogens, reproductive organ development, and temperature reactions [20,21,22]. *ASHR3* plays a key role in adjusting vernalization, stamen and root development, and pathogen defense [23,24,25]. In summary, *ASH1* family genes play important roles in regulating growth and development and enhancing resistance to biotic and abiotic stresses in plants. Despite these reports, studies regarding the involvement of *ASH1* family genes in tolerance to extreme temperatures and regulating plant development in response to temperature remain limited.

Cotton (*Gossypium* spp.) is the world’s most important cash crop; its fibers can be used as a textile material, and its seeds are a source of oil and vegetable protein [26]. The basal requirements for seasonal temperature during cotton growth limit the global area of arable land available for cotton cultivation [27]. In recent years, the global climate has been gradually warming, and the number of extreme weather events has increased [28]. Additionally, some planting areas are prone to “cold springs,” which severely affect cotton growth and development at the seedling stage [29]. Mining candidate genes associated with tolerance to high and low temperatures is critical for molecular improvement efforts that aim to increase the adaptability of cotton to temperature stress. Although many *ASH1* family members have recently been reported to be associated with plant growth, development, and tolerance to adverse stress, the identification and functional analysis of *ASH1* genes in upland cotton (*Gossypium hirsutum* L.) are still limited. In this study, *GhASH1*s were identified in upland cotton, and the evolution of the *ASH1* gene family was analyzed in different species. Subsequently, *GhASHH1.A* and *GhASHH2.A*, which were differentially expressed under high- and low-temperature stress, were screened from the transcriptome data. To investigate their impact on ambient temperature and flowering, *GhASHH1.A* and *GhASHH2.A* expression were silenced by VIGS to examine their roles in regulating low- and high-temperature resistance and flowering time. These findings provide genetic resources for future breeding of early-maturing and temperature-stress-tolerant cotton varieties.

## 2. Results

### 2.1. Identification of ASH1 Family Genes and Phylogenetic Analysis in Plants

**Figure 1:**
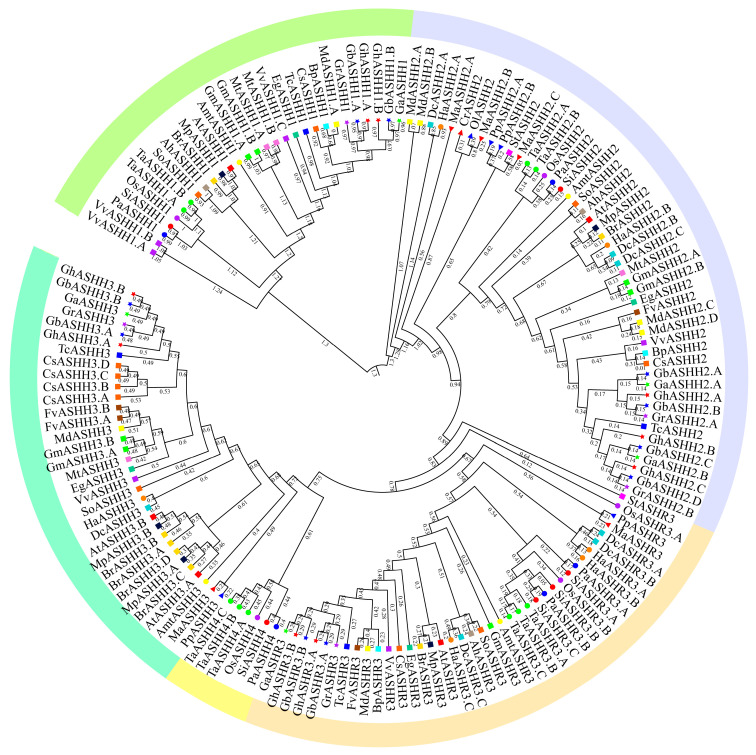
Phylogenetic tree of ASH1 proteins in green plants. The right triangles represent algae, the left triangles represent bryophytes, the circles represent dicotyledonous plants, the squares represent monocotyledonous plants, and the triangles represent cotton. The evolutionary tree is divided into five groups, each represented by a different color: turquoise, ASHH1; Purple, ASHH2; Orange, ASHR3; Yellow, ASHH4; and Cyan, ASHH3.

To comprehensively study their origin and evolution, we identified ASH1 proteins across 30 different species. The results revealed a total of 155 ASH1 proteins in 30 species, and nine GhASH1 proteins were detected in *G. hirsutum* (Table S1). The number of *ASH1* proteins in each species was relatively small (usually less than 10); these proteins first appeared in algae, and the number of *ASH1*s increased with the evolution of plants (Figure S1). More *ASH1* family genes were identified in monocotyledonous and eudicot plants than in lower plants; only one *ASH1* protein exists in algae (*Chlamydomonas reinhardtii* and *Ostreococcus lucimarinus*), whereas bryophytes exhibit four (*Physcomitrium patens*) and five (*Marchantia polymorpha*) ASH1s. All angiosperms had more than three *ASH1*s: monocot species had five to ten *ASH1*s, and eudicot species had three to ten *ASH1*s. The tree topology of *ASH1*s was well supported in most branches with five main groups (*ASHH1*, *ASHH2*, *ASHR3*, *ASHH3*, and *ASHH4*). *ASHH2* represents the ancestral group of *ASH1*s, and *ASHH1* was absent in algae and bryophytes, presenting only in monocotyledonous and eudicot plants. *ASHH3* was found only in eudicot plants but not in bryophytes or monocotyledonous plants. *ASHH4* and *ASHR3* were first identified in bryophytes (Figure 1). Furthermore, specific branches were observed in both monocotyledons and dicotyledons, which could be attributed to gene duplication events occurring after the differentiation of monocotyledons or gene loss events following the formation of evolutionary branches.

Phylogenetic analysis revealed that *ASH1*s in cacao and cotton clustered together and were more closely related to each other than were those in other plants. Specifically, six genes in *Gossypium* clustered within the same branch, consisting of two from the diploid cotton species *G. arboreum* and *G. raimondii*, two homologous *ASH1*s from *G. hirsutum*, and two homologous *ASH1*s from *G. barbadense*. However, in one branch of the ASHH2 subclass, *G. hirsutum* had only one homologous gene (*GhASHH2.C*), which may have been caused by the absence of the *ASH1* gene across the evolution of plants. During the morphological evolution of green plants, as plant morphology becomes more complex, the size of the genome tends to increase. To investigate this trend, we conducted calculations on the ratio of the number of *ASH1* family genes to that of the entire genome (Figure S2). Our findings revealed a relatively stable ratio of *ASH1*s to total genome genes.

### 2.2. Conserved Domain and Motif Analysis for the ASH1 Family

**Figure 2:**
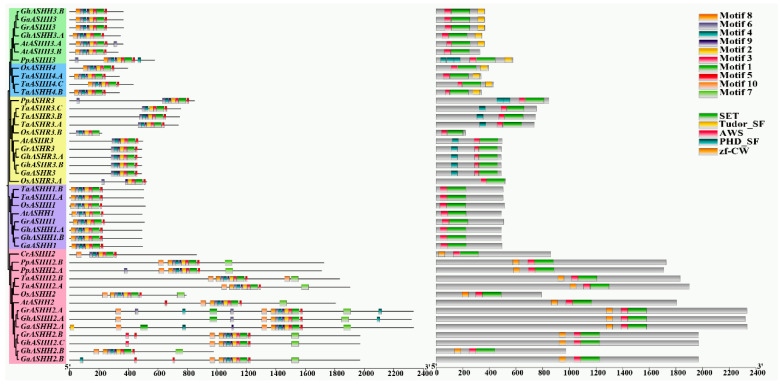
Comparison of the conserved motifs and conserved domains of *ASH1s*. The ten motifs are displayed in rectangles of different colors and are annotated in Figure S2. Domain composition of *ASH1*. The green, yellow, pink, blue, and orange rectangles represent the SET, Tudor_SF, AWS, PHD_SF, and zf-CW domains, respectively.

The conserved domain and motif composition of *ASH1* were determined by comparing the protein sequences of different types of representative plants and three *Gossypium* species. These were clearly clustered into five groups, which was consistent with the above results (Figure 2). Regarding motif analysis, we selected ten motifs for analysis to understand the structural and functional characteristics of the ASH1 proteins in different species, which were highly conserved among and within subgroups of the ASH1 protein family. In terms of the distribution of motifs, most *ASH1s* belonging to the same branch presented a similar motif composition, despite the deletion of motifs in individual proteins. The *ASHH1* branch genes had eight motifs, and most *ASHH2* branch members contained ten motifs, whereas *CrASHH2* contained eight motifs. Motif 2 and motif 7 began to appear during the process of bryophyte evolution. The *ASHR3* branch genes had six or eight motifs. The *ASHH3* and *ASHH4* branch genes had eight motifs, except *AtASHH3.A* and *PpASHH4* with nine and ten motifs, respectively. Like the *Arabidopsis* ASH1 protein domain, ASH1 proteins of all species have only two typically conserved AWS domains and SET domains. In addition, the *ASHH2* branch contains a zf-CW domain, some *ASHH3* branches contain a PHD domain, and the *ASHH3* and *ASHH4* branches contain a Tudor_SF domain. These results suggest that *GhASH1* gene family members clustered in the same group may share similar motifs and structural features.

### 2.3. Chromosomal Distribution and Collinearity of the GhASH1 Gene Family

**Figure 3:**
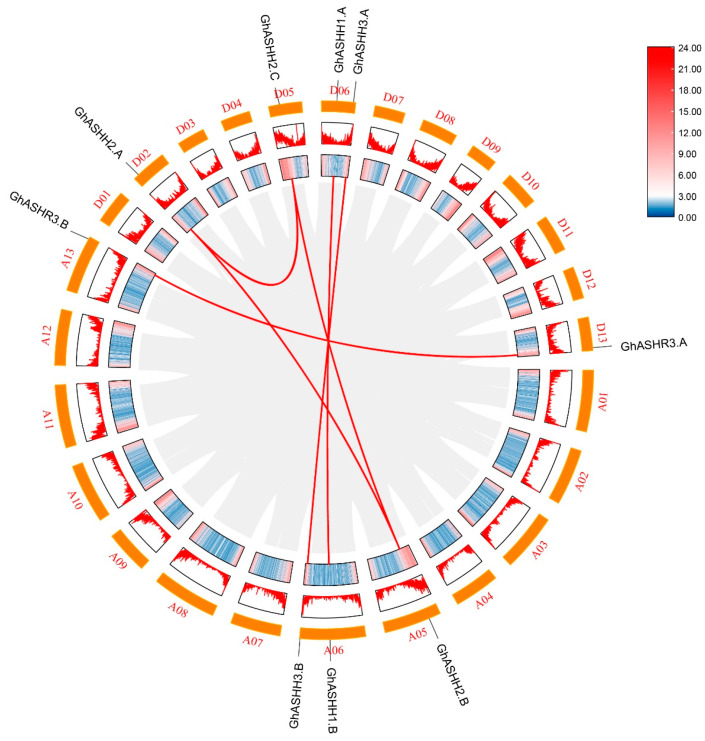
Duplication analysis of *ASH1s* within *G. hirsutum*. Duplications of *GhASH1s* on the chromosome. The gene ID on each chromosome corresponds to the approximate location of each *ASH1* gene.

The nine *GhASH1* members are distributed across seven chromosomes: the A06 and D06 chromosomes contain the most *GhASH1s* (two members), and A05, A13, D02, D15, and D13 each contain one *GhASH1* gene (Figure 3). Additionally, to identify gene replication events among members of the *GhASH1* gene family, six homologous duplicated gene pairs were discovered. To better understand the evolutionary constraints controlling the functional divergence of the *GhASH1* gene family, in the *G. hirsutum* genome, the Ka/Ks values of all duplicates were calculated to be lower than 1 (Figure S3). These results demonstrate the origination of orthologs and paralogs from whole genome duplication (WGD) before polyploidization during the time course of evolution.

### 2.4. Cis-Elements and GO Analysis of GhASH1s

**Figure 4:**
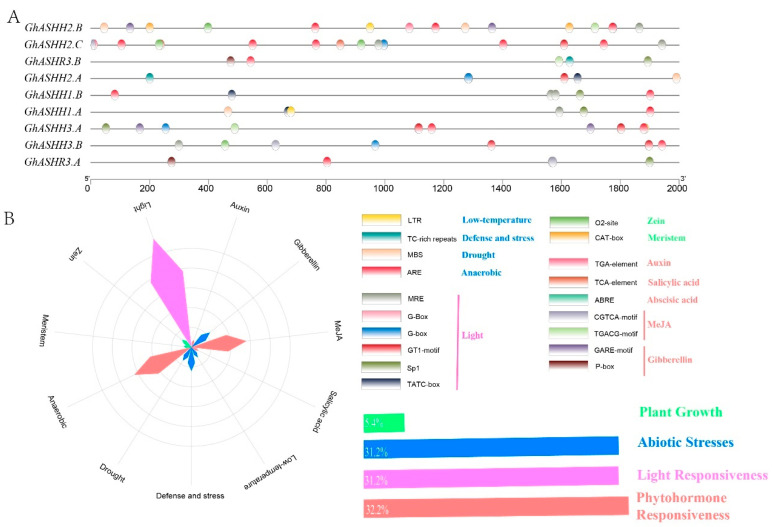
Analysis of cis-acting elements of the *ASH1* subfamily. (**A**) *ASH1* cis-acting element distribution; the cis-elements in the *GhASH1s* are marked by different colors. (**B**) Statistics regarding the number of cis-acting elements in *ASH1*; blue histograms, stress responses; red histograms, plant hormone regulation; green histograms, growth and development; and pink histograms, light responsiveness. The proportions of distinct cis-elements in each of the four categories.

The prediction of cis-acting regulatory elements can provide clues to gene expression patterns in plants subjected to tissue or environmental stress [30]. We predicted the potential homeopathic regulatory elements of *ASH1*s in upland cotton through the 2 kb upstream region in the PlantCare database. A total of 19 cis-regulatory elements, including growth and development promoters and hormones, light, and abiotic stress response elements, were detected (Figure 4A). The largest was the light-responsive element, which contained 32.2% predictive cis-elements. The plant hormone response and abiotic stress response elements ranked second, accounting for 31.2% each (Figure 4B). Among the hormone response elements, MeJA (CGTCA motif, TGACG motif) accounted for the largest proportion, followed by gibberellin response elements (GARE motif, P box). In the category of abiotic stress responses, elements related to oxygen deficiency-induced AREs were the most common, followed by defense and stress response elements (TC-rich repeats). The proportion of growth regulatory factors was minimal, and only two cis-acting factors were predicted. Interestingly, various cis-acting regulatory elements were widely distributed on the promoter of *GhASH1*, and *GhASH1* may be critical in the regulation of cotton development and stress resistance.

**Figure 5:**
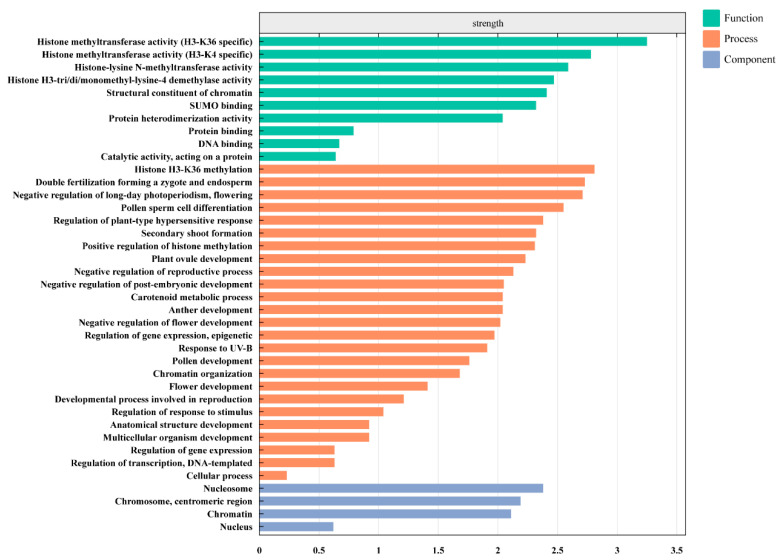
*ASH1* family GO analysis; green represents molecular functions, orange represents biological processes, and purple represents cellular components.

GO analysis was performed using *Arabidopsis* protein sequences as a reference, with the potential involvement of GhASH1 proteins in numerous biological processes, cellular components, and molecular processes. Moreover, we found that the molecular function processes mainly involved the activation of histone methylation transferases (H3K36 and H3K4), and the biological processes involved the methylation of histone H3K36, negative regulation of long-day photoperiodism, flowering, and regulation of the response to stimuli. Among the cell components, the nucleosome, chromosome, and centromeric regions were the main ones involved (Figure 5). Interestingly, ASHs mainly interact with nucleosomes and function by regulating histone methylation.

### 2.5. Expression Patterns of ASH1s Under Different Abiotic Stresses and in Different Tissues

**Figure 6:**
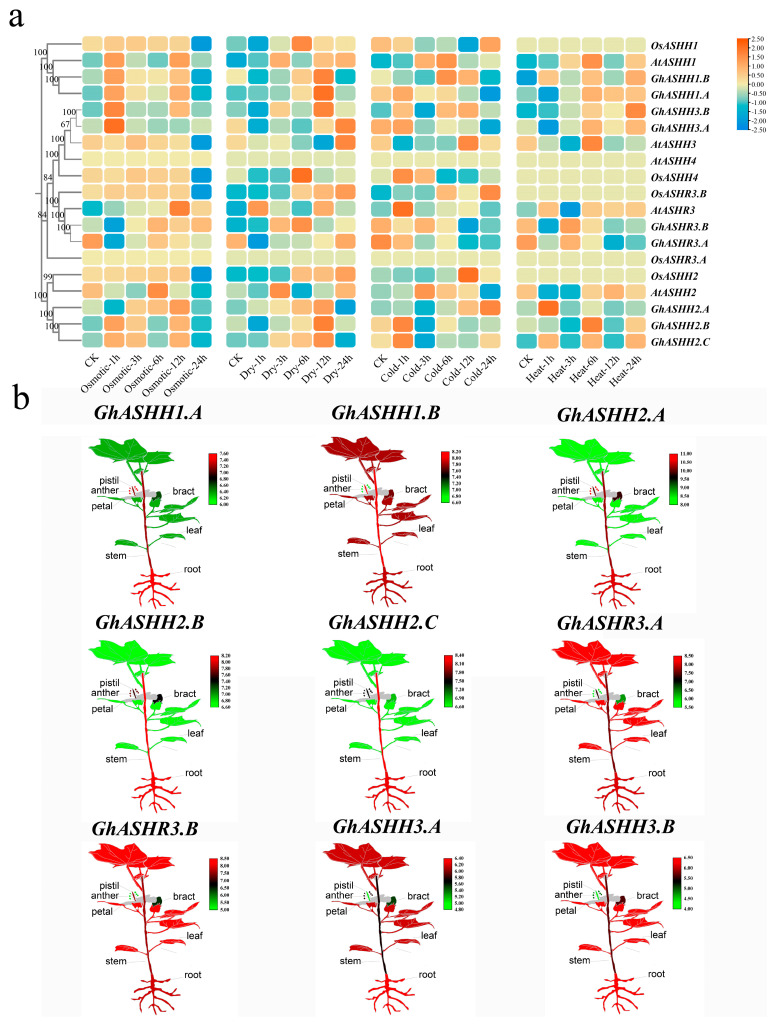
Expression patterns of *ASH1s* under different abiotic stresses. (**a**) The color scales with Z scores beside the heatmap indicate gene expression levels. Low and high transcript abundances are indicated by blue and red, respectively. (**b**) Differential expression of representative *GhASH1* genes in different tissues.

Plants are susceptible to various environmental stresses during their growth cycle, and gene expression patterns are usually closely related to the function of genes. Therefore, we studied the expression of *ASH1* under four kinds of stress in the model plants *A. thaliana* and rice together with *G. hirsutum* (Figure 6a). The results revealed that homologous genes presented the same expression trends in different species; among them, *ASHH1* expression increased after 1 h of osmotic stress and then decreased to different degrees. Additionally, *ASH1* expression differed under different stress conditions. The expression level of *GhASHH1.A* gradually decreased with increasing low-temperature stress duration, whereas the expression level increased with increasing high-temperature stress duration. Even under similar stress conditions, the expression of different *ASH1*s differed. The expression level of *GhASHH1.A* decreased with increasing low-temperature stress duration, whereas the expression level of *GhASHH2.A* increased with increasing low-temperature stress. Furthermore, *GhASH1* has diverse expression levels across multiple tissues (Figure 6b); *GhASHH1.A* and *GhASHH2.A* expression were upregulated in flowering organs, indicating that these genes may be involved not only in temperature stress but also in the regulation of plant development.

**Figure 7:**
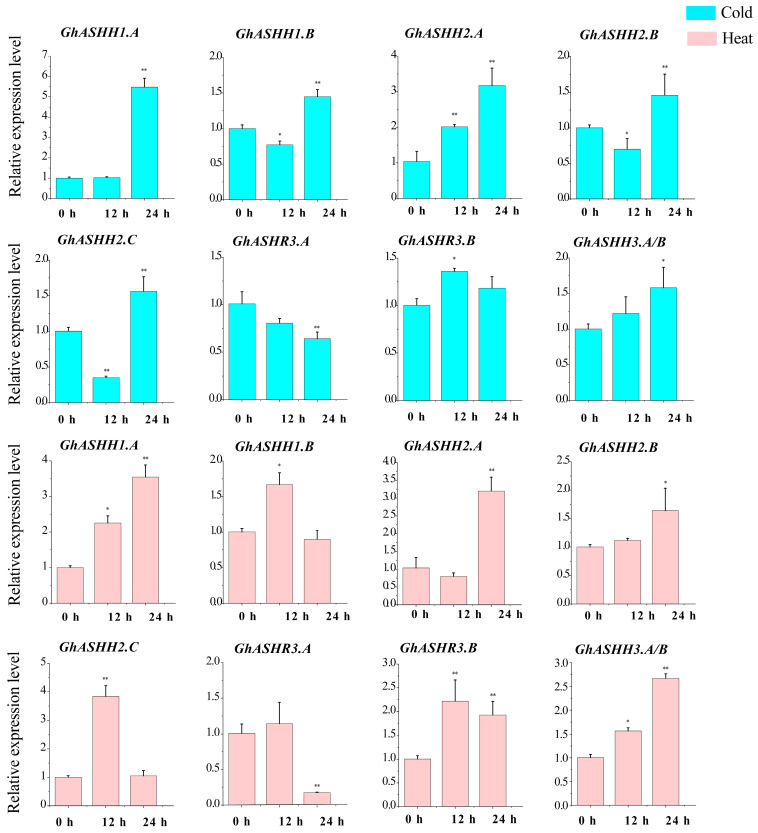
The expression levels of eight GhASH1s under cold and heat stress. The data for three biological replicates are represented by error bars, which show the standard deviations. Blue and pink represent cold and heat stress, respectively. The asterisks indicate significant differences according to Student’s *t* test. *, *p* < 0.05; **, *p* < 0.01.

To further investigate the role of *ASH1S*, we selected 8 *GhASH1* genes for qRT–PCR analysis (because the homology of *GhASHH3.A* and *GhASHH3.B* sequences was greater than 99%, we could not design specific primers for differentiation). Overall, the eight candidate *GhASH1* genes were highly responsive to both cold and heat (Figure 7). In addition to *GhASHR3.A* and *GhASHR3.B*, the other 6 *GhASH1* genes were highly responsive to cold stress. Under heat stress, except for *GhASHR3.B*, the expression level of the other 7 *GhASH1s* tested after heat stress was greater than that of the control. In addition, the expression levels of *GhASH1.A* and *GhASH2.A* significantly increased under both high-temperature and low-temperature stress. Taken together, these findings suggest that *GhASH1s* may be involved in the resistance of cotton to temperature stress.

### 2.6. Silencing of GhASHH1.A and GhASHH2.A Compromises the Temperature Tolerance of Cotton to Stress

**Figure 8:**
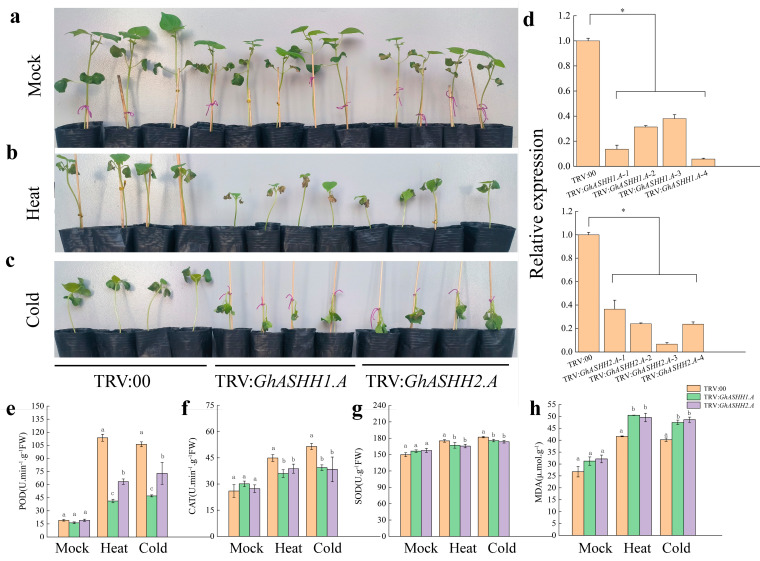
Validation of *GhASHH1.A* and *GhASHH2.A* silencing via the VIGS technique. (**a**–**c**) Effects of mock (25 °C), heat (42 °C), and cold (12 °C) stress on TRV:00, TRV:*GhASHH1.A*, and TRV:*GhASHH2.A* phenotypes. (**d**) Relative expression levels of exogenous *GhASHH1.A* and *GhASHH2.A* in VIGS-treated plants grown to the three-leaf stage under LD conditions. (**e**–**h**) Physiological and biochemical indices of TRV:00, TRV:*GhASHH1.A* and TRV:*GhASHH2.A* plants subjected to different stress treatments. SOD activity, POD activity, CAT activity, and MDA content of TRV:00, TRV:*GhASHH1.A* and TRV:*GhASHH2.A* plants. The experiment was conducted in triplicate, and the data were analyzed via the Student’s *t* test. The different letters (a, b, and c) indicate significant differences according to Duncan’s honestly significant difference test. The asterisks indicate significant differences according to Student’s *t* test. *, *p* < 0.05.

To investigate the function of *GhASHH1.A* and *GhASHH2.A* under temperature stress, we used VIGS technology to silence *GhASHH1.A* and *GhASHH2.A* expression. After 9 days, the leaves of the TRV:*GhCLA*-positive control plants demonstrated a leaf bleaching phenotype, and we treated the TRV:00 plants and the TRV:*GhASHH1.A*- and TRV:*GhASHH2.A*-silenced plants at different temperatures (25 °C, 42 °C, and 12 °C) (Figure 8a–c). The results revealed that the leaves of TRV:*GhASHH1.A* and TRV:*GhASHH2.A* plants turned yellow faster and lost more water than those of the TRV:00 plants under high-temperature stress. Under low-temperature stress, TRV:*GhASHH1.A* and TRV:*GhASHH2.A* plants wilted significantly compared with TRV:00 plants. These results indicate that *GhASHH1.A* and *GhASHH2.A* expression had a positive effect on plant response to heat and cold conditions.

To investigate the effects of abiotic stress on *GhASHH1.A* and *GhASHH2.A* expression, we evaluated the changes in the activities of SOD, POD, and CAT in TRV:*GhASHH1.A*- and TRV:*GhASHH2.A*-silenced cotton plants (Figure 8e–g). The results showed that the POD, SOD, and CAT activities in the TRV:*GhASHH1.A* and TRV:*GhASHH2.A* plants treated at 25 °C were not significantly greater than those in the TRV:00 plants. Under cold and heat stress, the POD, SOD, and CAT activities of the TRV:*GhASHH1.A* and TRV:*GhASHH2.A* plants were significantly lower than those of the TRV:00 plants. Conversely, a notable increase in the MDA content was detected (Figure 8h). A decrease in POD activity in TRV:*GhASHH1.A* plants was more obvious than that in TRV:*GhASHH2.A* plants. These results indicate that the silencing of the *GhASHH1.A* and *GhASHH2.A* genes decreased the stress resistance of cotton plants. We suggest that *GhASHH1.A* and *GhASHH2.A* expression might positively regulate tolerance to temperature stress by regulating the activity of antioxidant enzymes and eliminating ROS.

### 2.7. Silencing of GhASHH1.A and GhASHH2.A Expression Affected High-Temperature-Induced Flowering

In order to investigate whether *GhASHH1.A* and *GhASHH2.A* participate in the Thermomorphogenesis of plants, after the albino phenotype of TRV:*GhCLA* leaves appeared, to determine the effect of suppressed transcription of the target genes on plant traits, plants whose transcript levels were reduced by half were selected for target silencing. Plants whose transcript levels were reduced by half were selected for target silencing. Phenotypic analysis was performed separately on plants subjected to different temperatures at the seedling stage. After 10 days of treatment at different temperatures, the growth rates of the silenced plants were lower than those of the TRV:00 plants. Under the 32 °C treatment, plant growth accelerated, and the stem diameter, fresh weight, and leaf number were greater than those under the 25 °C treatment (Figure S4).

**Figure 9:**
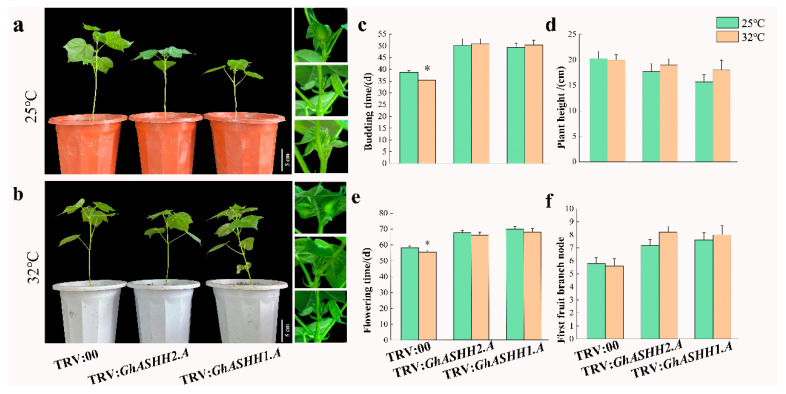
Budding time statistics of TRV:00 and gene-silenced plants under different treatments. (**a**) Budding properties of TRV:00, TRV:*GhASHH1.A*, and TRV:*GhASHH2.A* plants after 10 days at 25 °C. (**b**) Budding properties of TRV:00, TRV:*GhASHH1.A*, and TRV:*GhASHH2.A* plants after 10 days at 32 °C. (**c**) Statistics regarding bud emergence times of the empty vector and gene-silenced plants after different temperature treatments. (**d**) Height statistics of the empty vector and gene-silenced plants after different temperature treatments. (**e**) Statistics regarding the flowering times of the empty vector and gene-silenced plants after different temperature treatments. (**f**) First fruit branch node statistics of the empty carrier and gene-silenced plants after different temperature treatments. The asterisks indicate significant differences according to Student’s *t* test. *, *p* < 0.05.

To investigate whether gene silencing affects high-temperature-induced flowering, time of bud emergence, flowering time, and initial nodes of fruit branches were subsequently analyzed under different temperature treatments (Figure 9a,b). The budding and flowering times of the gene-silenced plants were delayed by 9 and 10 days, respectively, at 25 °C, and the number of initial nodes of fruiting branches increased. The budding and flowering times of the TRV:00 plants at 32 °C were 2–3 d earlier than those at 25 °C, whereas the budding and flowering times of the gene-silenced plants did not differ (Figure 9c–f). In summary, *GhASHH1.A* and *GhASHH2.A* might positively regulate flowering and affect plant responses to ambient temperature changes.

**Figure 10:**
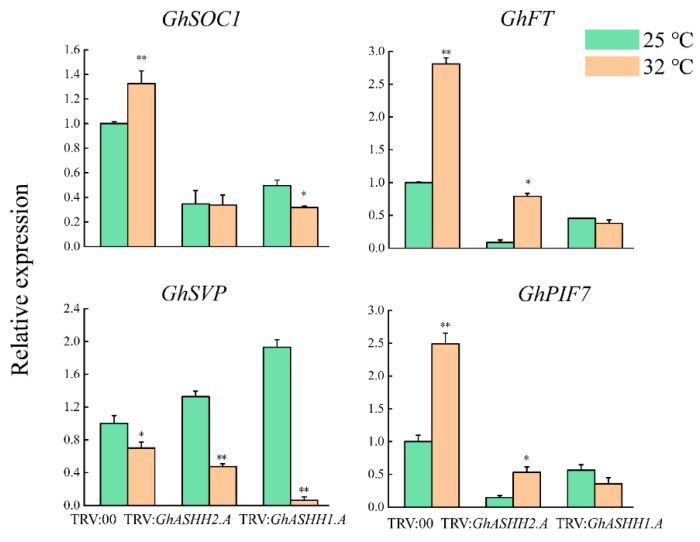
The temperature pathway regulates the relative expression of flowering-related genes. The asterisks indicate significant differences according to Student’s *t* test. *, *p* < 0.05; **, *p* < 0.01.

To understand the response of *GhASHH1.A* and *GhASHH2.A* in the temperature regulation of flowering, the expression patterns of the flower development-related genes *GhSOC1 (SUPPRESSOR OF OVEREXPRESSION OF CONSTANS 1)*, *GhFT (FLOWERING LOCUS T)*, *GhSVP (SHORT VEGETATIVE PHASE*), and *GhPIF7* (*PHYTOCHROME Interaction Factor 7*) in response to temperature regulation were investigated (Figure 10). The expression of *GhSOC1* and *GhFT* in the gene-silenced plants decreased after treatment at 25 °C and 32 °C. In addition, as a flowering inhibitor, *GhSVP* expression decreased at 32 °C, indicating that it may also respond to the regulation of plant flowering by high temperature. *GhPIF7* is an important gene in plant development whose expression is induced by high temperature, and it was highly expressed in TRV:00 plants but expressed at low levels in gene-silenced plants at 32 °C, demonstrating the same trend as that of *GhSOC1* and *GhFT*. Taken together, these findings suggest that *GhASHH1.A* and *GhASHH2.A* might regulate flowering by influencing the expression levels of key genes in the temperature pathway (Figure S5).

## 3. Discussion

Upland cotton, an excellent antistress pioneer crop, shows good adaptability in harsh environments [31]. However, subjected to an abnormal global climate, the growth of cotton is also being tested in terms of temperature. Seedling stress has an important effect on the growth and quality of cotton [32]. The *ASH1* gene family, as important family members of the TrxG complex, participates in the activity of histone H3K36 methyltransferase and has been shown in many plants to induce plant tolerance to abiotic stress and regulate growth and development [33].

### 3.1. Evolution of the ASH1 Gene Family in Plants

With the widespread adoption of whole-genome sequencing, the *ASH1* family, which plays crucial roles in plant development toward flowering, plant disease resistance, and various abiotic stresses, has been extensively identified and studied [33,34,35,36,37]. We conducted a comprehensive analysis of *ASH1* gene families across different lineages of plants to investigate their evolutionary origins. A total of 155 *ASH1* family genes were identified across 30 plants spanning algae, bryophytes, monocotyledonous plants, and dicotyledonous plants. The *ASH1* gene was initially discovered in algae. The *ASHH2* gene cluster is likely to represent the ancestral form of the *ASH1* gene family, while an increase in the number of *ASH1* genes can be observed in bryophytes. This could be attributed to two rounds of genome-wide replication events that occurred in bryophytes [38,39]. However, the absence of both *ASHH1* and *ASHH3* gene clusters in both bryophytes and gymnosperms suggests that this branch emerged during angiosperm evolution. Interestingly, both monocotyledonous and dicotyledonous plant members among basal angiosperms presented distinct clustering patterns. Monocotyledonous plant members clustered within the *ASHH4* branch whereas dicotyledonous plant members clustered within the *ASHH3* branch. Notably, this divergence indicates potential differences between monocotyledonous and dicotyledonous plant genes. According to the phylogenetic tree and the chromosome localization and collinear analysis data, many fragments of the *ASH1* family in upland cotton may have been derived from genome-wide duplication events during different stages of plant evolution. Therefore, the *ASH1* family is an ancient gene family, and its members were retained in the upland cotton genome through WGD, chromosome fragment repetition, and other amplification methods and then differentiated into different functions.

Over the course of evolution, the *ASH1* gene family has undergone significant expansion and has diversified into five major branches in land plants. Specifically, angiosperms have experienced remarkable amplification of *ASH1* family genes, resulting in the formation of endemic clades in dicotyledonous and monocotyledonous plants. This phenomenon can be attributed to polyploidization events that occurred in at least 70% of angiosperms throughout their evolutionary history, serving as a primary driving force for alterations in plant genome size [40]. Genome duplication plays a pivotal role in facilitating the expansion of the *ASH1* family. Compared with closely related species, species that have undergone WGD present relatively greater numbers of *ASH1*s, particularly *G. hirsutum*, *G. barbadense*, and wheat, which display even greater amplification within this gene family. Notably, almost all instances of copy number increase events observed on the molecular evolutionary tree correspond to known whole-genome events [38]. The proportion of *ASH1*s across the entire genome remains stable across angiosperms, indicating that WGD contributes significantly more to gene family expansion than other forms of gene duplication [41].

### 3.2. Evolution and Functional Diversity of the Cotton ASH1 Gene Family

Previous studies have shown that the *ASH1* gene contains SET and Associated with SET (AWS) domains, which are required for the catalytic function of H3K36 and are involved in the plant response to external environmental influences [42]. We selected five different types of representative plants and three *Gossypium* species to analyze their conserved gene domains and motifs and found different domains in different branches, indicating that different gene clusters have different functions. *ASHR3* members have an additional PHD domain near the N-terminus, and *ASHR3* has catalytic effects on H3K36me1 and possibly H3K36me2, which are involved in regulating cell division competence in the root meristem [24]. *ASHH1* knockout in *A. thaliana* decreased H3K4me3 and H3K36me3 abundance at the *SOC1* locus, and *SDG708/OsASHH1* knockout also resulted in a genome-wide decrease in H3K36me1/2/3 abundance in rice during early growth stages [43]. *ASHH2* contains an additional CW (cysteine and tryptophan conserved) domain at the N-terminus as a member of the histone modification reader module for epigenetic regulation, which can be found in at least five other protein families in higher plants. *Arabidopsis ASHH2/SDG8* acts as the major H3K36me2/3 writer [44,45]. Predictably, cotton harbors a similar function.

Cis-acting elements regulate gene transcription to enable plants to respond to environmental development [46]. Previous studies have shown that gene expression is regulated by various signals, such as endogenous plant hormones including auxin (IAA), abscisic acid (ABA), brassinolide (BR), osmotic stress, salt, cold, heat, and drought, which are related to various response elements in the promoter region [47]. Analysis of *ASH1* family cis-acting elements revealed that the promoter region of *GhASH1s* contains a variety of hormone response elements and abiotic stress response elements (AREs, MBSs, and LTRs), indicating that *ASH1* gene expression is influenced by the external environment. In addition, through GO analysis of the biological functions of the *ASH1* gene family, many biological processes related to plant growth and development and the environmental response were identified. GO analysis revealed that the main function of *GhASH1s* is to regulate histone H3K36 methylation, which plays an irreplaceable role across the entire growth cycle of plants [18]. However, functional studies of *GhASH1s* in upland cotton are still lacking; therefore, their possible functions can be predicted by analyzing their functions.

Transcriptome data analysis revealed that the *GhASH1* gene family was highly expressed mainly in leaves and reproductive organs. This finding is similar to those of previous studies in which *ASH1*s were found to be involved in the regulation of flower formation in plants [20,43,48]. We also analyzed transcriptome data regarding several abiotic stresses, including drought, osmotic stress, and high-temperature and low-temperature treatments, revealing the differential expression of *ASH1s* under these external signals, and homologous genes presented the same expression trend in the model plants *A. thaliana*, rice, and upland cotton. *ASHH2* positively regulates drought resistance in *A. thaliana* and rice [21,33], which is consistent with the transcriptome data, suggesting that upland cotton may have similar functions. These results indicate that the *GhASH1* family is widely involved in nonbiological stress responses and growth and development processes in cotton plants; among them, *GhASHH1.A* and *GhASHH2.A* may be key candidate genes. In future research, we can focus on these gene family members, which are highly important for exploring their potential biological functions.

### 3.3. GhASHH1.A- and GhASHH2.A-Silenced Plants in Response to Temperature Stress and Temperature-Controlled Flowering

Previous studies have shown that *ASH1* homologs play a key role in abiotic stress responses and plant growth and development. To further study the role of *GhASH1s* in the temperature stress response and temperature regulation development of cotton, *GhASHH1.A* and *GhASHH2.A* expressions were knocked out via the TRV virus knock down approach system. The results showed that at 12 °C and 42 °C, the death and wilt rates of *GhASHH1.A*- and *GhASHH2.A*-silenced plants were greater than those of the control plants. Elimination of the *GhASHH1.A* and *GhASHH2.A* genes decreased the stress resistance of cotton plants and increased their susceptibility to temperature stress. Under normal temperature, the redox state in plants is balanced; when seedlings are subjected to temperature stress, a large amount of ROS is induced, and the activity of antioxidant enzymes in plants increases to remove excess ROS [31]. However, when the gene-silenced plants were subjected to temperature stress because gene silencing could not control the synthesis of antioxidants in the body, the plants died, and the MDA content in the body also increased.

In *A. thaliana*, *ashh1*-mutant plants presented a late-flowering phenotype, and *ashh2*-mutant plants presented an early-flowering phenomenon [20]. However, our study revealed that *GhASHH1.A* and *GhASHH2.A*-silenced plants all presented a late-flowering phenotype, possibly because *ASHH2* is involved in regulating *FLC* chromatin deposition in *A. thaliana* to regulate flowering. However, upland cotton is a subtropical crop that enjoys warmth and light; its photoperiod sensitivity is lost with selection and domestication, and its *FLC* gene is missing [49], which is similar to the results of the *ashh2*-mutant in rice [43]. It has been previously reported that *GhASHH1.A* and *GhASHH2.A* can respond to temperature stress. In addition, *ASHH1* is reportedly involved in the regulation of plant flowering and the response to Spring [19]. Thermomorphogenesis is the response mechanism of plants to temperature, and high temperatures below a heat stress temperature cause changes in plant morphology, such as hypocotyl and petiole elongation and early flowering [50,51]. The flowering and development of upland cotton are highly dependent on temperature, but there has been little research on the regulation and development of cotton in response to temperature changes. The optimal temperature of the cotton seedling stage is 20–30 °C, but the temperature of the environment often fluctuates with climate change and cannot reach the optimal conditions [52]; therefore, we chose 32 °C as the relatively high temperature. Pajoro et al. reported that reduced deposition of H3K36me3 in *Arabidopsis* resulted in temperature-induced changes in alternative splicing genes [53]. *ASH1* genes in upland cotton may also be involved in the regulation of temperature-induced flowering.

An increase in ambient temperature can promote early flowering of plants [54], so the flowering of TRV:00 plants after high-temperature treatment occurred earlier than that of the control, whereas early flowering of gene-silenced plants was not induced by high temperature. We selected and examined the expression levels of genes involved in the temperature pathway regulation of flowering genes, including *GhSOC1*, *GhFT*, *GhSVP*, and *GhPIF7*, in TRV:00 and TRV:*GhASHH1.A*- and TRV:*GhASHH2.A*- silenced plants under different temperature treatments. Our results revealed that the expression levels of *GhSOC1* and *GhFT* were significantly reduced in the gene-silenced plants, suggesting that *GhASHH1.A* and *GhASHH2.A* may regulate flowering by regulating these key genes. After high-temperature treatment, the expression levels of *GhSOC1*, *GhFT*, and *GhPIF7* in the TRV:00 plants significantly increased, whereas the expression levels of *GhSVP* significantly decreased, indicating that high temperature induced the expression of *GhPIF7* in the TRV:00 plants and then regulated the expression of *GhSOC1* and *GhFT* to promote the early flowering of the TRV:00 plants. However, in the gene-silenced plants, *GhASHH1.A*- and *GhASHH2.A*-silencing prevented the plants from responding to temperature or weakened their ability to respond to temperature, so they did not participate in the regulation of flowering time. Studies have shown that heat stress can upregulate the expression of genes related to chlorophyll biosynthesis and promote the expression of *SOC1* to induce early flowering of *A. thaliana* [55]. In upland cotton, *GhSOC1-1* activates flower transformation in response to high temperature, which is the dominant factor in the induction and control of flowering in cotton in response to high temperature and short sunshine in subtropical regions [56]. In addition, it has been reported that relatively high temperatures can inhibit the expression of *SVP* and the binding of *SVP* and the *FT* promoter, thus promoting the early flowering of plants and reducing the expression of *GhSVP* at high temperatures [57,58]. *FT* chromatin can be recognized by the H3K4/H3K36 methylation reader MRG1/2 protein, which regulates the circadian rhythm of the *FT* gene and plays a role in plant flowering by integrating flowering signals from different pathways [59]. *PIF7*, a core transcription factor in high-temperature signal transduction, is involved in histone methylation to promote flowering [60]. It has been speculated that *GhASHH1.A* and *GhASHH2.A* are involved in the cotton response to temperature regulation by regulating *PIF7* and *FT*. Overall, these results suggest that *GhASHH1.A* and *GhASHH2.A* positively regulate flowering time and may be involved in flowering in response to temperature.

## 4. Materials and Methods

### 4.1. Identification and Sequence Analysis of ASH1 Family Members

On the basis of the IDs of *ASH1* family members reported in the literature, protein sequences encoded by five *ASH1* genes in *A. thaliana* were downloaded from the TAIR website as query sequences. In the Phytozome plant database (https://phytozome-next.jgi.doe.gov/, accessed on 10 March 2024), we BLAST searched for similar sequences in the protein sequences of 29 species. The SMART database model (http://smart.embl-heidelberg.de/, accessed on 15 March 2024) was used to verify each ASH1 protein, and the sequence containing the SET domain (PF00856) and the AWS domain (PF17907) was assumed to be *ASH1* [61].

### 4.2. Phylogenetic Analysis, Conserved Domain Analysis, and Motif Analysis of ASH1s

Multiple sequence alignment of 155 ASH1 protein sequences was performed using MEGA 13 software. The phylogenetic tree was subsequently constructed via the 155 ASH1 protein sequence file, and the bootstrap value was set to 1000 by using the adjacency method. The original tree was visualized via an interactive tree of life (iTOL) (https://itol.embl.de/, accessed on 19 March 2024) [30,62]. Dicots, monocots, and model plants were selected as outgroup species; gene conserved domains were downloaded from the NCBI Conserved Domains Database (CDD) (https://www.ncbi.nlm.nih.gov/cdd/, accessed on 1 April 2024), and conserved motif analysis was performed via MEME (https://meme-suite.org/meme/index.html, accessed on 25 March 2024).

### 4.3. Chromosomal Location and Collinear Analysis of the Cotton ASH1 Gene Family

The genomic locations of members of the ASH1 gene family were obtained from the genome annotation file of *G. hirsutum*. The collinearities of ASH1s among upland cotton plants were evaluated via TBtools MCScanx software v1.116. The chromosomal locations and collinearities were visualized with Circos software v1.116. The homologous relationships and nonsynonymous substitution rates (Ka) and synonymous substitution rates (Ks) of homologous ASH1s of *G. hirsutum* were determined via TBtools software v1.116 [63].

### 4.4. Transcriptomic Expression Analysis

RNA-seq data regarding different tissues and stresses of the *G. hirsutum* accession were downloaded from Zhejiang University (http://cotton.zju.edu.cn/, accessed on 4 April 2024). *A. thaliana* and rice stress RNA-seq data were obtained via the Arabidopsis eFP Browser (https://www.arabidopsis.org/, accessed on 4 April 2024) and the Transcriptome ENcyclopedia of Rice (https://tenor.dna.affrc.go.jp/, accessed on 5 April 2024). TBtools was used to construct a heatmap for visualization. The 2.0 kb upstream region of the GhASH1 family nucleotide sequence promoter was extracted, and the PlantCARE database (http://bioinformatics.psb.ugent.be/, accessed on 6 April 2024) was used for screening. GO (Gene Ontology) enrichment analysis was performed with STRING [30].

### 4.5. Vector Construction and Procedure for VIGS and Expression Profiling

For the VIGS experiment, approximately 300–500 bp nucleotide sequences of *GhASHH1.A* and *GhASHH2.A* were designed via NCBI Primer-BLAST (https://blast.ncbi.nlm.nih.gov/Blast.cgi/, accessed on 15 March 2024). The fragments obtained were then inserted into the pTRV2 vector to create pTRV2: *GhASHH1.A* and *GhASHH2.A* constructs. The VIGS experiment was performed as previously described [64]. The qRT-PCR expression analysis of selected genes was conducted using the designed primers (Table S2). The housekeeping gene Ghact was used as an internal control, and relative expression levels were calculated via the 2^−ΔΔCT^ method [65].

### 4.6. Experimental Materials and Treatments

After bleaching the TRV *CLV1* plants, empty TRV:00 carrier plants and silent plants were grown to 2 weeks of age and then subjected to a high temperature (42 °C) or a low temperature (12 °C). After 10 days of temperature stress treatment, the activities of antioxidant enzymes (CAT, POD, and SOD) and the content of MDA were determined. The nitroblue tetrazole photoreduction method was employed to measure superoxide dismutase (SOD) activity; the guaiacol colorimetric method was used to assess peroxidase (POD) activity; and a colorimetric method was employed to determine the malonic dialdehyde (MDA) concentration in plant tissues. Throughout the measurement period, three technical replicates were used for each sample. After the bleaching of the TRV:*CLV1* plants, the empty carrier plants and TRV:00-silenced plants were cultured in incubators at 25 °C and 32 °C, respectively, and then were transplanted at room temperature after 10 days of treatment to observe their properties.

## 5. Conclusions

In this study, we selected 30 plants for genome-wide identification of their *ASH1* genes and analyzed their evolution, dividing them into 5 different clades on the basis of their sequence homology and evolutionary trees. The *ASH1* gene has a similar gene structure, motif distribution, and composition in the same group, and there are differences among different groups. We analyzed the cis-acting elements and GO terms associated with *ASH1* in upland cotton and revealed that *GhASH1* not only participates in stress and defense responses but also plays an important role in growth and development. The function of *GhASHH1.A* and *GhASHH2.A* was further identified by VIGS, suggesting that *GhASHH1.A*- and *GhASHH2.A*-silencing may reduce the ability of cotton to respond to temperature stress and affect high-temperature-induced flowering.

## Data Availability

All the data generated or analyzed during this study are included in this article and its Supplementary Data Files.

## Supplementary Materials

The supporting information can be downloaded at https://www.mdpi.com/article/10.3390/ijms252011321/s1.

## References

[1] Patel D., Franklin K.A. (2009). Temperature-regulation of plant architecture. Plant Signal. Behav..

[2] Capovilla G., Schmid M., Posé D. (2015). Control of flowering by ambient temperature. J. Exp. Bot..

[3] Zhu T., De Lima C., De Smet I. (2021). The Heat is On: How Crop Growth, Development and Yield Respond to High Temperature. J. Exp. Bot..

[4] Kidokoro S., Shinozaki K., Yamaguchi-Shinozaki K. (2022). Transcriptional regulatory network of plant cold-stress responses. Trends Plant Sci..

[5] Sunkar R., Chinnusamy V., Zhu J., Zhu J.K. (2007). Small RNAs as big players in plant abiotic stress responses and nutrient deprivation. Trends Plant Sci..

[6] Pérez-Clemente R.M., Vives V., Zandalinas S.I., López-Climent M.F., Muñoz V., Gómez-Cadenas A. (2013). Biotechnological approaches to study plant responses to stress. BioMed Res. Int..

[7] Lahiri A., Venkatasubramani P.S., Datta A. (2019). Bayesian modeling of plant drought resistance pathway. BMC Plant Biol..

[8] Li Z.-G., Ding X.-J., Du P.-F. (2013). Hydrogen sulfide donor sodium hydrosulfide-improved heat tolerance in maize and involvement of proline. J. Plant Physiol..

[9] Sanchez M.d.l.P., Aceves-García P., Petrone E., Steckenborn S., Vega-León R., Álvarez-Buylla E.R., Garay-Arroyo A., García-Ponce B. (2015). The impact of Polycomb group (PcG) and Trithorax group (TrxG) epigenetic factors in plant plasticity. New Phytol..

[10] Cheng K., Lei C., Zhang S., Zheng Q., Wei C., Huang W., Xing M., Zhang J., Zhang X., Zhang X. (2023). Genome-wide identification and characterization of polycomb repressive complex 2 core components in upland cotton (*Gossypium hirsutum* L.). BMC Plant Biol..

[11] Pu L., Sung Z.R. (2015). PcG and trxG in plants—Friends or foes. Trends Genet..

[12] Bieluszewski T., Xiao J., Yang Y., Wagner D. (2021). PRC2 activity, recruitment, and silencing: A comparative perspective. Trends Plant Sci..

[13] Kleinmanns J.A., Schubert D. (2014). Polycomb and Trithorax group protein-mediated control of stress responses in plants. Biol. Chem..

[14] Bastow R., Mylne J.S., Lister C., Lippman Z., Martienssen R.A., Dean C. (2004). Vernalization requires epigenetic silencing of FLC by histone methylation. Nature.

[15] Ramakrishnan M., Zhang Z., Mullasseri S., Kalendar R., Ahmad Z., Sharma A., Liu G., Zhou M., Wei Q. (2022). Epigenetic stress memory: A new approach to study cold and heat stress responses in plants. Front. Plant Sci..

[16] Ornelas-Ayala D., Cortés-Quiñones C., Olvera-Herrera J., García-Ponce B., Garay-Arroyo A., Álvarez-Buylla E.R., Sanchez M.d.l.P. (2022). A Green Light to Switch on Genes: Revisiting Trithorax on Plants. Plants.

[17] Tanaka Y., Katagiri Z.-I., Kawahashi K., Kioussis D., Kitajima S. (2007). Trithorax-group protein ASH1 methylates histone H3 lysine 36. Gene.

[18] Schmähling S., Meiler A., Lee Y., Mohammed A., Finkl K., Tauscher K., Israel L., Wirth M., Philippou-Massier J., Blum H. (2018). Regulation and function of H3K36 di-methylation by the trithorax-group protein complex AMC. Development.

[19] Zeng J., Yang L., Tian M., Xie X., Liu C., Ruan Y. (2023). SDG26 Is Involved in Trichome Control in *Arabidopsis thaliana*: Affecting Phytohormones and Adjusting Accumulation of H3K27me3 on Genes Related to Trichome Growth and Development. Plants.

[20] Liu B., Berr A., Chang C., Liu C., Shen W.-H., Ruan Y. (2016). Interplay of the histone methyltransferases SDG8 and SDG26 in the regulation of transcription and plant flowering and development. Biochim. Biophys. Acta (BBA) Gene Regul. Mech..

[21] Sun X., Chen L., Su Y. (2021). Histone methyltransferase SDG8 in dehydration stress. J. Univ. Sci. Tech..

[22] Berr A., McCallum E.J., Alioua A., Heintz D., Heitz T., Shen W.-H. (2010). Arabidopsis histone methyltransferase SET DOMAIN GROUP8 mediates induction of the jasmonate/ethylene pathway genes in plant defense response to necrotrophic fungi. Plant Physiol..

[23] De-La-Peña C., Rangel-Cano A., Alvarez-Venegas R. (2012). Regulation of disease-responsive genes mediated by epigenetic factors: Interaction of Arabidopsis–Pseudomonas. Mol. Plant Pathol..

[24] Thorstensen T., Grini P.E., Mercy I.S., Alm V., Erdal S., Aasland R., Aalen R.B. (2008). The Arabidopsis SET-domain protein ASHR3 is involved in stamen development and interacts with the bHLH transcription factor ABORTED MICROSPORES (AMS). Plant Mol. Biol..

[25] Kumpf R., Thorstensen T., Rahman M.A., Heyman J., Nenseth H.Z., Lammens T., Herrmann U., Swarup R., Veiseth S.V., Emberland G. (2014). The ASH1-RELATED3 SET-Domain protein controls cell division competence of the meristem and the quiescent center of the *Arabidopsis* primary root. Plant Physiol..

[26] Wang C., Liu J., Xie X., Wang J., Ma Q., Chen P., Yang D., Ma X., Hao F., Su J. (2023). GhAP1-D3 positively regulates flowering time and early maturity with no yield and fiber quality penalties in upland cotton. J. Integr. Plant Biol..

[27] Zhao H., Chen Y., Liu J., Wang Z., Li F., Ge X. (2023). Recent advances and future perspectives in early-maturing cotton research. New Phytol..

[28] Zhang B., Gao H., Wang G., Zhang S., Shi M., Li Y., Huang Z., Xiang W., Gao W., Zhang C. (2022). Guvermectin, a novel plant growth regulator, can promote the growth and high temperature tolerance of maize. Front. Plant Sci..

[29] Hossain E., Zhang Z., Dong W., Wang S., Liu M., Liu E., Mei X. (2022). Plastic Film Mulching Improved Maize Yield, Water Use Efficiency, and N Use Efficiency under Dryland Farming System in Northeast China. Plants.

[30] Letunic I., Bork P. (2019). Interactive Tree Of Life (iTOL) v4: Recent updates and new developments. Nucleic Acids Res..

[31] Ling P., Ju J., Zhang X., Wei W., Luo J., Li Y., Hai H., Shang B., Cheng H., Wang C. (2024). The Silencing of *GhPIP5K2* and *GhPIP5K22* Weakens Abiotic Stress Tolerance in Upland Cotton (*Gossypium hirsutum*). Int. J. Mol. Sci..

[32] Li H.-M., Liu S.-D., Ge C.-W., Zhang X.-M., Zhang S.-P., Chen J., Shen Q., Ju F.-Y., Yang Y.-F., Li Y. (2019). Association Analysis of Drought Tolerance and Associated Traits in Upland Cotton at the Seedling Stage. Int. J. Mol. Sci..

[33] Chen K., Du K., Shi Y., Yin L., Shen W., Yu Y., Liu B., Dong A. (2021). H3K36 methyltransferase SDG708 enhances drought tolerance by promoting abscisic acid biosynthesis in rice. New Phytol..

[34] Zhang X., Menard R., Li Y., Coruzzi G.M., Heitz T., Shen W.H., Berr A. (2020). Arabidopsis SDG8 Potentiates the Sustainable Transcriptional Induction of the Pathogenesis-Related Genes PR1 and PR2 During Plant Defense Response. Front. Plant Sci..

[35] Yadav C.B., Muthamilarasan M., Dangi A., Shweta S., Prasad M. (2016). Comprehensive analysis of SET domain gene family in foxtail millet identifies the putative role of SiSET14 in abiotic stress tolerance. Sci. Rep..

[36] Jian H., Wei F., Chen P., Hu T., Lv X., Wang B., Wang H., Guo X., Ma L., Lu J. (2023). Genome-wide analysis of SET domain genes and the function of GhSDG51 during salt stress in upland cotton (*Gossypium hirsutum* L.). BMC Plant Biol..

[37] Lee S., Fu F., Xu S., Lee S.Y., Yun D.-J., Mengiste T. (2016). Global Regulation of Plant Immunity by Histone Lysine Methyl Transferases. Plant Cell.

[38] Gao B., Chen M., Li X., Liang Y., Zhang D., Wood A.J., Oliver M.J., Zhang J. (2022). Ancestral gene duplications in mosses characterized by integrated phylogenomic analyses. J. Syst. Evol..

[39] One Thousand Plant Transcriptomes Initiative (2019). One thousand plant transcriptomes and the phylogenomics of green plants. Nature.

[40] Li Z., Li M., Wang J. (2022). Asymmetric subgenomic chromatin architecture impacts on gene expression in resynthesized and natural allopolyploid Brassica napus. Commun. Biol..

[41] Soltis P.S., E Soltis D. (2016). Ancient WGD events as drivers of key innovations in angiosperms. Curr. Opin. Plant Biol..

[42] Ng D.W.-K., Wang T., Chandrasekharan M.B., Aramayo R., Kertbundit S., Hall T.C. (2007). Plant SET domain-containing proteins: Structure, function and regulation. Biochim. Biophys. Acta (BBA) Gene Struct. Expr..

[43] Liu B., Wei G., Shi J., Jin J., Shen T., Ni T., Shen W.H., Yu Y., Dong A. (2016). SET DOMAIN GROUP 708, a histone H3 lysine 36-specific methyltransferase, controls flowering time in rice (*Oryza sativa*). New Phytol..

[44] Huang Y., Jiang L., Liu B.-Y., Tan C.-F., Chen D.-H., Shen W.-H., Ruan Y. (2019). Evolution and conservation of polycomb repressive complex 1 core components and putative associated factors in the green lineage. BMC Genom..

[45] Liu Y., Liu S., Zhang X., Liang X., Zahid K.R., Liu K., Liu J., Deng L., Yang J., Qi C. (2016). Structure and Function of CW Domain Containing Proteins. Curr. Protein Pept. Sci..

[46] Nakashima K., Yamaguchi-Shinozaki K., Shinozaki K. (2014). The transcriptional regulatory network in the drought response and its crosstalk in abiotic stress responses including drought, cold, and heat. Front. Plant Sci..

[47] Tang K., Dong C.-J., Liu J.-Y. (2016). Genome-Wide Comparative Analysis of the Phospholipase D Gene Families among Allotetraploid Cotton and Its Diploid Progenitors. PLoS ONE.

[48] Berr A., Shafiq S., Pinon V., Dong A., Shen W. (2015). The trxG family histone methyltransferase SET DOMAIN GROUP 26 promotes flowering via a distinctive genetic pathway. Plant J..

[49] Tian Y., Zhang T. (2021). MIXTAs and phytohormones orchestrate cotton fiber development. Curr. Opin. Plant Biol..

[50] Hou Y., Yan Y., Cao X. (2022). Epigenetic regulation of thermomorphogenesis in Arabidopsis thaliana. aBIOTECH.

[51] He K., Cao X., Deng X. (2021). Histone methylation in epigenetic regulation and temperature responses. Curr. Opin. Plant Biol..

[52] Snider J.L., Thangthong N., Pilon C., Virk G., Tishchenko V. (2018). OJIP-fluorescence parameters as rapid indicators of cotton (*Gossypium hirsutum* L.) seedling vigor under contrasting growth temperature regimes. Plant Physiol. Biochem..

[53] Pajoro A., Severing E., Angenent G.C., Immink R.G.H. (2017). Histone H3 lysine 36 methylation affects temperature-induced alternative splicing and flowering in plants. Genome Biol..

[54] Liu J., Feng L., Gu X., Deng X., Qiu Q., Li Q., Zhang Y., Wang M., Deng Y., Wang E. (2019). An H3K27me3 demethylase-HSFA2 regulatory loop orchestrates transgenerational thermomemory in Arabidopsis. Cell Res..

[55] Wang Z., Shen Y., Yang X., Pan Q., Ma G., Bao M., Zheng B., Duanmu D., Lin R., Larkin R.M. (2019). Overexpression of particular MADS-box transcription factors in heat-stressed plants induces chloroplast biogenesis in petals. Plant Cell Environ..

[56] Ma L., Yan Y. (2022). GhSOC1s Evolve to Respond Differently to the Environmental Cues and Promote Flowering in Partially Independent Ways. Front. Plant Sci..

[57] Marín-González E., Matías-Hernández L., Aguilar-Jaramillo A.E., Lee J.H., Ahn J.H., Suárez-López P., Pelaz S. (2015). SHORT VEGETATIVE PHASE Up-Regulates TEMPRANILLO2 Floral Repressor at Low Ambient Temperatures. Plant Physiol..

[58] Lee J.H., Ryu H.-S., Chung K.S., Posé D., Kim S., Schmid M., Ahn J.H. (2013). Regulation of temperature-responsive flowering by MADS-box transcription factor repressors. Science.

[59] Guo Z., Li Z., Liu Y., An Z., Peng M., Shen W.H., Dong A., Yu Y. (2020). MRG1/2 histone methylation readers and HD2C histone deacetylase associate in repression of the florigen gene FT to set a proper flowering time in response to day-length changes. New Phytol..

[60] Bianchimano L., De Luca M.B., Borniego M.B., Iglesias M.J., Casal J.J. (2023). Temperature regulation of auxin-related gene expression and its implications for plant growth. J. Exp. Bot..

[61] El-Gebali S., Mistry J., Bateman A., Eddy S.R., Luciani A., Potter S.C., Qureshi M., Richardson L.J., Salazar G.A., Smart A. (2019). The Pfam protein families database in 2019. Nucleic Acids Res..

[62] Larkin M.A., Blackshields G., Brown N.P., Chenna R., McGettigan P.A., McWilliam H., Valentin F., Wallace I.M., Wilm A., Lopez R. (2007). Clustal W and Clustal X version 2.0. Bioinformatics.

[63] Chen C.J., Chen H., Zhang Y., Thomas H.R., Frank M.H., He Y.H., Xia R. (2020). TBtools: An Integrative Toolkit Developed for Interactive Analyses of Big Biological Data. Mol. Plant.

[64] Gao W., Long L., Zhu L.-F., Xu L., Gao W.-H., Sun L.-Q., Liu L.-L., Zhang X.-L. (2013). Proteomic and virus-induced gene silencing (VIGS) analyses reveal that gossypol, brassinosteroids, and jasmonic acid contribute to the resistance of cotton to *Verticillium dahliae*. Mol. Cell. Proteom..

[65] Livak K.J., Schmittgen T.D. (2001). Analysis of relative gene expression data using real-time quantitative PCR and the 2^−ΔΔ*C*T^ Method. Methods.

