# Supplementary material for: GhASHH1.A and GhASHH2.A Improve Tolerance to High and Low Temperatures and Accelerate the Flowering Response to Temperature in Upland Cotton (Gossypium hirsutum)
Source: Int J Mol Sci. 2024 Oct 21;25(20):11321. doi: 10.3390/ijms252011321 (PMC11508336; doi:10.3390/ijms252011321)
Supplement: Supplementary file 1 [file ijms-25-11321-s001.zip › Supplement.pdf]

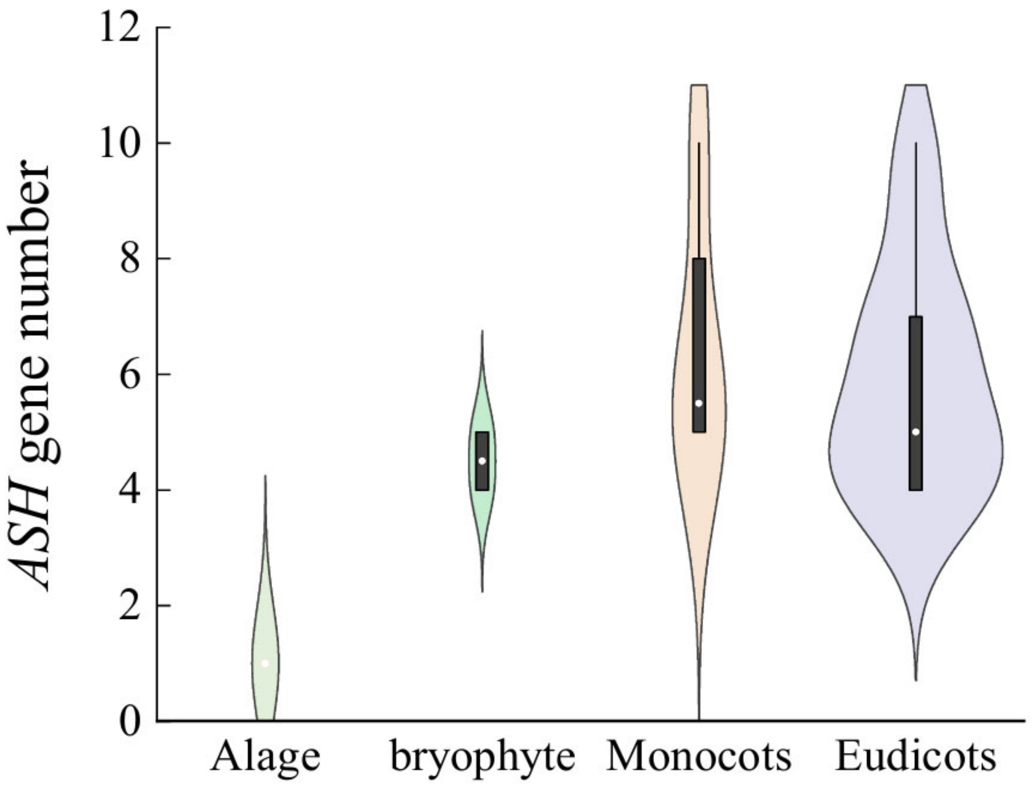

Figure S1. Evolutionary analysis of ASH1 family. Violin plot of ASH1 gene number for different categories of plants.

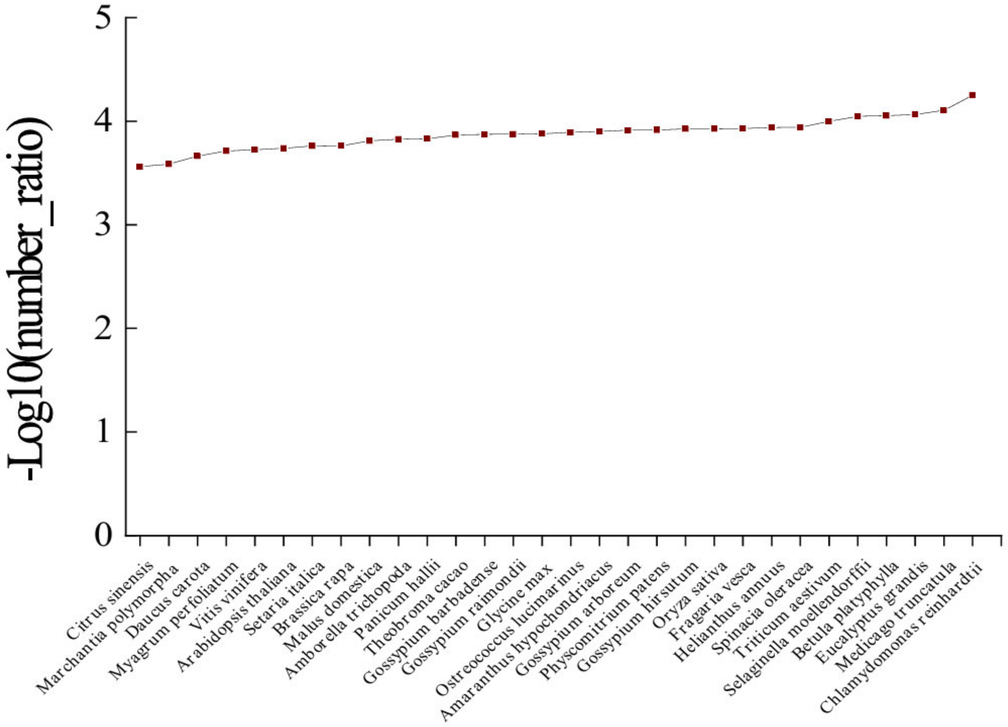

Figure S2 .The -log10 (number ratio) of ASH1 family genes in each plant compared with whole-genome genes.

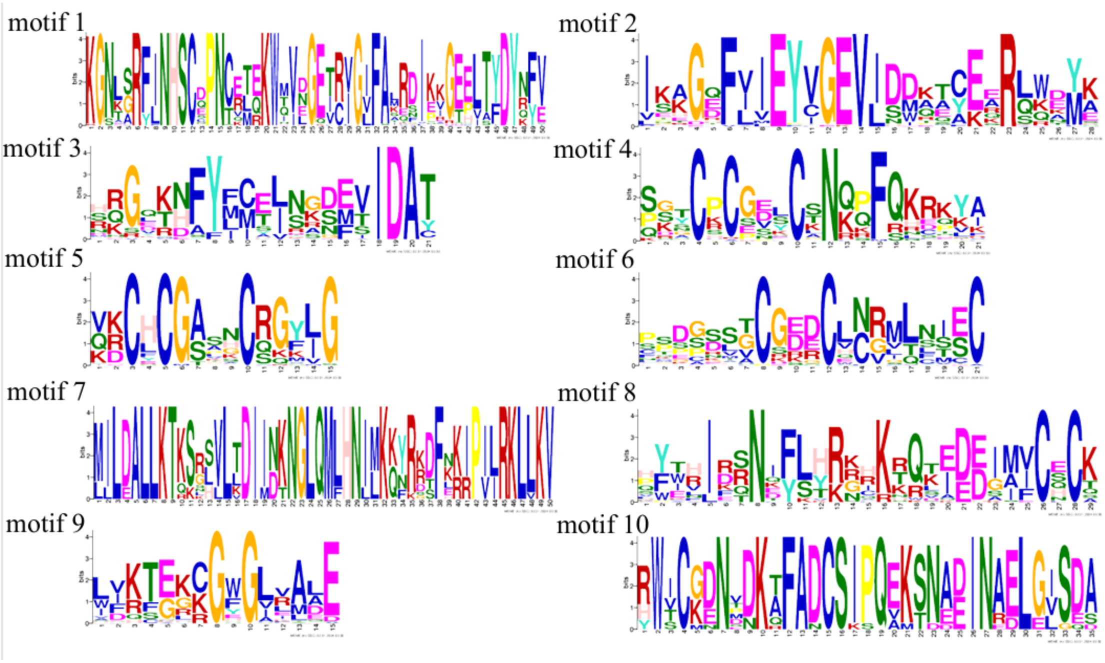

Figure S3. Base composition of each motif.

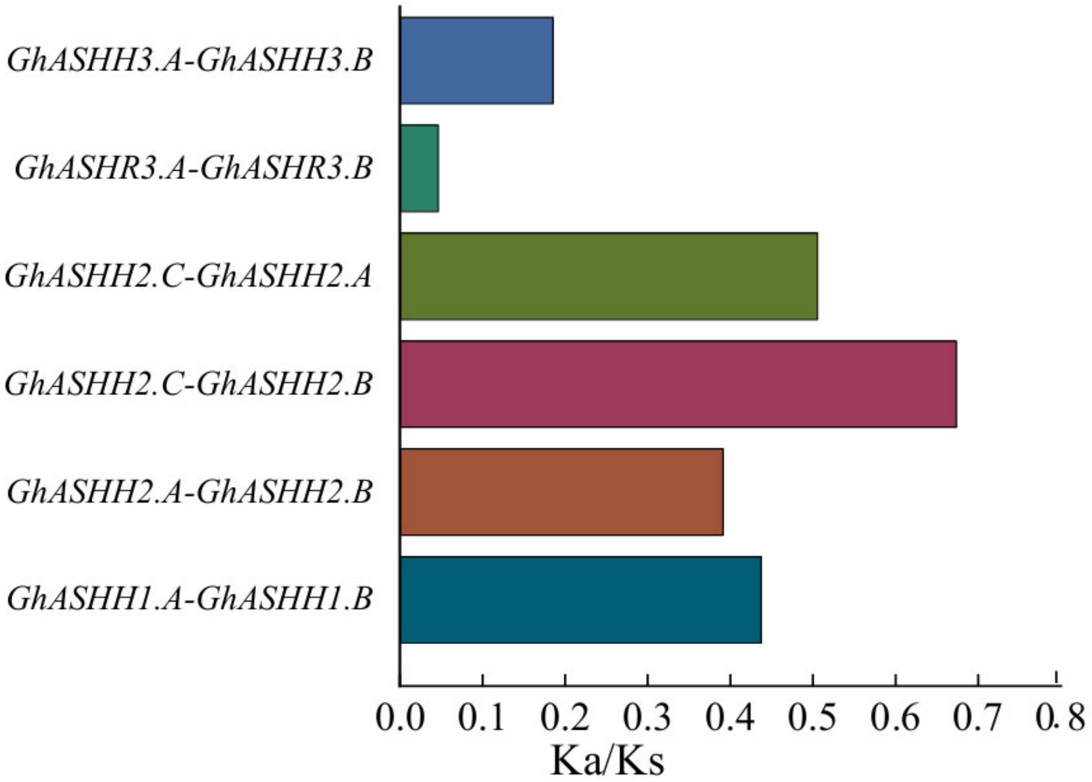

Figure S4. Schematic diagram of non-synonymous (Ka) to synonymous (Ks) ratio in cotton.

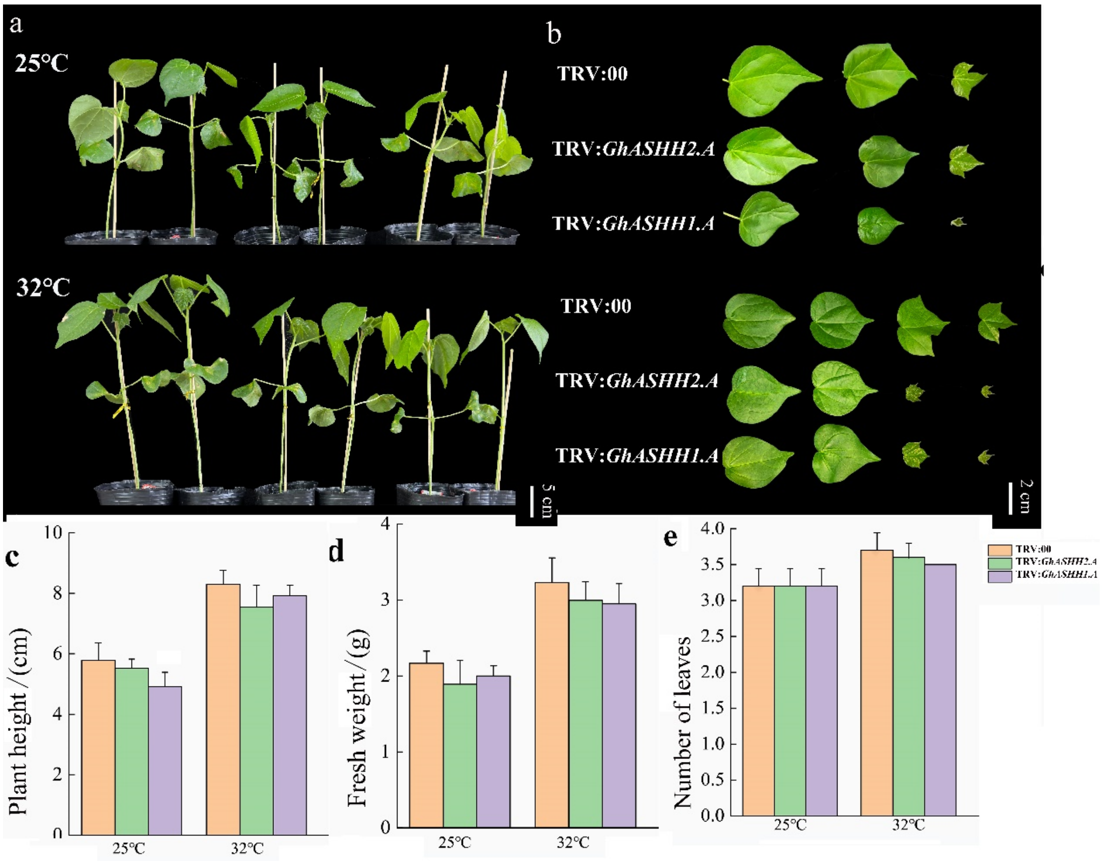

Figure S5. The silencing of *GhASHH1.A* and *GhASHH2.A* slowed down the growth of cotton. a. Phenotypes of TRV:00, TRV:*GhASHH1.A* and TRV:*GhASHH2.A* after 10 days at different temperatures. b. Leaf phenotypes of TRV:00, TRV:*GhASHH1.A* and TRV:*GhASHH2.A* after 10 days at different temperatures. c. Plant height statistics of empty carrier and silenced gene at seedling stage under different treatments. d. Fresh weight statistics of empty carrier and silenced gene at seedling stage under different treatments. e. Number of leaves statistics of empty carrier and silenced gene at seedling stage under different treatments.
